# A centrosymmetric monoclinic polymorph of *N* ^1^,*N* ^4^-bis­(pyridin-3-yl­methyl­idene)benzene-1,4-diamine

**DOI:** 10.1107/S1600536811030789

**Published:** 2011-08-06

**Authors:** Kwang Ha

**Affiliations:** a School of Applied Chemical Engineering, The Research Institute of Catalysis, Chonnam National University, Gwangju 500-757, Republic of Korea

## Abstract

The complete molecule of the title compound, C_18_H_14_N_4_, is generated by the application of a centre of inversion. The dihedral angle between the central benzene ring and the pyridine ring is 31.88 (7)°. In the crystal, mol­ecules are stacked in columns along the *c* axis and several inter­molecular π–π inter­actions are present between the six-membered rings, the shortest centroid–centroid distance being 3.937 (2) Å. The structure reported herein represents a centrosymmetric polymorph of the previously reported non-centrosymmetric (*P*2_1_) form [Kim *et al.* (2005 ▶). *Bull. Korean Chem. Soc.* **26**, 892–898].

## Related literature

For the crystal structure of *N* ^1^,*N* ^4^-bis­(pyridin-3-yl­methyl­ene)benzene-1,4-diamine, see: Kim *et al.* (2005 ▶). For the crystal structure of *N* ^1^,*N* ^4^-bis­(pyridin-2-yl­methyl­ene)benzene-1,4-diamine, see: Chanda *et al.* (2002 ▶); Ball *et al.* (2004 ▶).
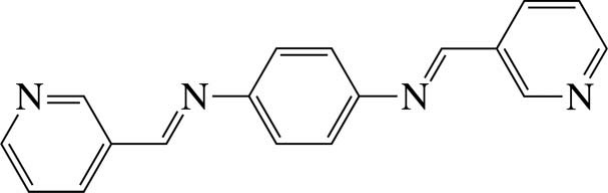


## Experimental

### 

#### Crystal data

C_18_H_14_N_4_
*M* *_r_* = 286.33
Monoclinic, 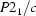

*a* = 16.622 (6) Å
*b* = 6.171 (2) Å
*c* = 7.159 (2) Å
β = 95.732 (8)°
*V* = 730.6 (4) Å^3^
*Z* = 2
Mo *K*α radiation
μ = 0.08 mm^−1^
*T* = 200 K
0.22 × 0.14 × 0.11 mm

#### Data collection

Bruker SMART 1000 CCD diffractometer
Absorption correction: multi-scan (*SADABS*; Bruker, 2000 ▶) *T*_min_ = 0.825, *T*_max_ = 1.000
5128 measured reflections
1790 independent reflections
1027 reflections with *I* > 2σ(*I*)
*R*_int_ = 0.049

#### Refinement

*R*[*F* ^2^ > 2σ(*F* ^2^)] = 0.053
*wR*(*F* ^2^) = 0.152
*S* = 1.03
1790 reflections
100 parameters
H-atom parameters constrained
Δρ_max_ = 0.21 e Å^−3^
Δρ_min_ = −0.24 e Å^−3^

### 

Data collection: *SMART* (Bruker, 2000 ▶); cell refinement: *SAINT* (Bruker, 2000 ▶); data reduction: *SAINT*; program(s) used to solve structure: *SHELXS97* (Sheldrick, 2008 ▶); program(s) used to refine structure: *SHELXL97* (Sheldrick, 2008 ▶); molecular graphics: *ORTEP-3* (Farrugia, 1997 ▶) and *PLATON* (Spek, 2009 ▶); software used to prepare material for publication: *SHELXL97*.

## Supplementary Material

Crystal structure: contains datablock(s) global, I. DOI: 10.1107/S1600536811030789/tk2772sup1.cif

Structure factors: contains datablock(s) I. DOI: 10.1107/S1600536811030789/tk2772Isup2.hkl

Supplementary material file. DOI: 10.1107/S1600536811030789/tk2772Isup3.cml

Additional supplementary materials:  crystallographic information; 3D view; checkCIF report

## Figures and Tables

**Fig. 1.**
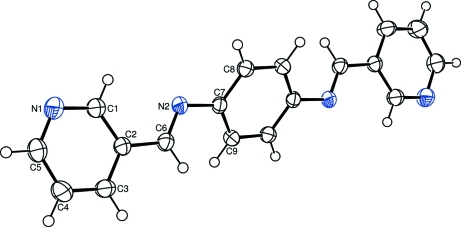
The structure of the title compound, with displacement ellipsoids drawn at the 50% probability level; H atoms are shown as small circles of arbitrary radius. Unlabelled atoms are related by the symmetry transformation: -x, -y, 1 - z.

**Fig. 2.**
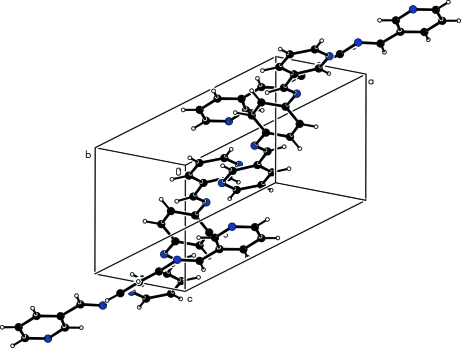
View of the unit-cell contents of the title compound.

## supplementary crystallographic information

### Comment

The title compound is a polydentate Schiff-base (Fig. 1), which can act as a monodentate or bis(monodentate) ligand, that is, one N atom or two N atoms of the pyridyl groups can coordinate to a metal ion or metal ions. The crystal structure of the this compound was previously reported in the non-centrosymmetric space group *P*2_1_ (Kim *et al.*, 2005). The centrosymmetric structure presented here is essentially the same as the published structure and represents a new monoclinic polymorph. In the present study the compound is isomorphous with the analogous compound *N*^1^,*N*^4^-bis(pyridin-2-ylmethylene)benzene-1,4-diamine (Chanda *et al.*, 2002; Ball *et al.*, 2004).

The asymmetric unit of the title molecule contains one half of the formula unit (Fig. 1); a centre of inversion is located in the midpoint of the compound, and therefore the two pyridyl rings are exactly parallel. The dihedral angle between the central benzene ring and the pyridine ring is 31.88 (7) °. The N2—C6/7 bond lengths and the C6—N2—C7 bond angle indicate that the imino N2 atom is *sp*^2^-hybridized [*d*(N2═C6) = 1.283 (2) Å and *d*(N2—C7) = 1.429 (2) Å; <C6—N2—C7 = 119.0 (2) °]. In the crystal structure, the molecules are stacked in columns along the *c* axis and several intermolecular π-π interactions are present between the six-membered rings, with a shortest centroid-centroid distance being 3.937 (2) Å (Fig. 2).

### Experimental

1,4-Phenylenediamine (1.0812 g, 9.998 mmol) and 3-pyridinecarboxaldehyde (2.1447 g, 20.023 mmol) in CH_3_CN (30 ml) were stirred for 3 h at room temperature. The precipitate was then separated by filtration, washed with CH_3_CN and ether, and dried under vacuum, to give a yellow powder (1.8899 g). Crystals suitable for X-ray analysis were obtained by slow evaporation from its ethylacetate solution.

### Refinement

H atoms were positioned geometrically and allowed to ride on their respective parent atoms [C—H = 0.95 Å and *U*_iso_(H) = 1.2*U*_eq_(C)].

### Crystal data

**Table:**

C_18_H_14_N_4_	*F*(000) = 300
*M**_r_* = 286.33	*D*_x_ = 1.301 Mg m^−^^3^
Monoclinic, *P*2_1_/*c*	Mo *K*α radiation, λ = 0.71073 Å
Hall symbol: -P 2ybc	Cell parameters from 1327 reflections
*a* = 16.622 (6) Å	θ = 2.5–28.2°
*b* = 6.171 (2) Å	µ = 0.08 mm^−^^1^
*c* = 7.159 (2) Å	*T* = 200 K
β = 95.732 (8)°	Plate, yellow
*V* = 730.6 (4) Å^3^	0.22 × 0.14 × 0.11 mm
*Z* = 2	

### Data collection

**Table:**

Bruker SMART 1000 CCD diffractometer	1790 independent reflections
Radiation source: fine-focus sealed tube	1027 reflections with *I* > 2σ(*I*)
graphite	*R*_int_ = 0.049
φ and ω scans	θ_max_ = 28.3°, θ_min_ = 2.5°
Absorption correction: multi-scan (*SADABS*; Bruker, 2000)	*h* = −22→19
*T*_min_ = 0.825, *T*_max_ = 1.000	*k* = −8→8
5128 measured reflections	*l* = −8→9

### Refinement

**Table:**

Refinement on *F*^2^	Primary atom site location: structure-invariant direct methods
Least-squares matrix: full	Secondary atom site location: difference Fourier map
*R*[*F*^2^ > 2σ(*F*^2^)] = 0.053	Hydrogen site location: inferred from neighbouring sites
*wR*(*F*^2^) = 0.152	H-atom parameters constrained
*S* = 1.03	*w* = 1/[σ^2^(*F*_o_^2^) + (0.064*P*)^2^ + 0.0036*P*] where *P* = (*F*_o_^2^ + 2*F*_c_^2^)/3
1790 reflections	(Δ/σ)_max_ < 0.001
100 parameters	Δρ_max_ = 0.21 e Å^−^^3^
0 restraints	Δρ_min_ = −0.24 e Å^−^^3^

### Special details

**Table:**

Geometry. All e.s.d.'s (except the e.s.d. in the dihedral angle between two l.s. planes) are estimated using the full covariance matrix. The cell e.s.d.'s are taken into account individually in the estimation of e.s.d.'s in distances, angles and torsion angles; correlations between e.s.d.'s in cell parameters are only used when they are defined by crystal symmetry. An approximate (isotropic) treatment of cell e.s.d.'s is used for estimating e.s.d.'s involving l.s. planes.
Refinement. Refinement of *F*^2^ against ALL reflections. The weighted *R*-factor *wR* and goodness of fit *S* are based on *F*^2^, conventional *R*-factors *R* are based on *F*, with *F* set to zero for negative *F*^2^. The threshold expression of *F*^2^ > σ(*F*^2^) is used only for calculating *R*-factors(gt) *etc*. and is not relevant to the choice of reflections for refinement. *R*-factors based on *F*^2^ are statistically about twice as large as those based on *F*, and *R*- factors based on ALL data will be even larger.

### Fractional atomic coordinates and isotropic or equivalent isotropic displacement parameters (Å^2^)

**Table:**

	*x*	*y*	*z*	*U*_iso_*/*U*_eq_	
N1	0.40327 (10)	0.2126 (3)	0.3182 (2)	0.0434 (5)	
N2	0.16109 (9)	0.0893 (2)	0.4230 (2)	0.0296 (4)	
C1	0.32643 (12)	0.1772 (3)	0.3548 (3)	0.0374 (5)	
H1	0.3091	0.0310	0.3622	0.045*	
C2	0.27031 (11)	0.3394 (3)	0.3825 (3)	0.0298 (5)	
C3	0.29604 (12)	0.5530 (3)	0.3732 (3)	0.0426 (6)	
H3	0.2602	0.6690	0.3922	0.051*	
C4	0.37490 (13)	0.5940 (3)	0.3356 (3)	0.0501 (6)	
H4	0.3938	0.7387	0.3282	0.060*	
C5	0.42572 (13)	0.4208 (3)	0.3092 (3)	0.0439 (6)	
H5	0.4795	0.4514	0.2831	0.053*	
C6	0.18734 (11)	0.2849 (3)	0.4232 (3)	0.0300 (5)	
H6	0.1520	0.3990	0.4507	0.036*	
C7	0.07963 (11)	0.0508 (3)	0.4617 (2)	0.0264 (4)	
C8	0.06382 (11)	−0.1381 (3)	0.5601 (2)	0.0293 (5)	
H8	0.1070	−0.2333	0.6006	0.035*	
C9	0.01465 (11)	0.1882 (3)	0.4004 (2)	0.0286 (4)	
H9	0.0243	0.3158	0.3318	0.034*	

### Atomic displacement parameters (Å^2^)

**Table:**

	*U*^11^	*U*^22^	*U*^33^	*U*^12^	*U*^13^	*U*^23^
N1	0.0308 (10)	0.0441 (11)	0.0568 (12)	0.0014 (8)	0.0123 (8)	0.0005 (8)
N2	0.0263 (9)	0.0297 (9)	0.0338 (8)	−0.0027 (7)	0.0080 (6)	0.0001 (7)
C1	0.0320 (12)	0.0327 (11)	0.0488 (12)	−0.0006 (8)	0.0108 (9)	0.0010 (9)
C2	0.0266 (10)	0.0296 (10)	0.0337 (10)	−0.0018 (8)	0.0057 (8)	−0.0005 (8)
C3	0.0331 (12)	0.0279 (11)	0.0688 (14)	0.0000 (9)	0.0149 (10)	−0.0012 (10)
C4	0.0388 (13)	0.0348 (12)	0.0790 (17)	−0.0103 (10)	0.0176 (11)	0.0000 (11)
C5	0.0288 (11)	0.0456 (13)	0.0586 (13)	−0.0069 (10)	0.0113 (10)	0.0003 (10)
C6	0.0261 (10)	0.0307 (11)	0.0338 (10)	0.0001 (8)	0.0060 (8)	−0.0016 (8)
C7	0.0248 (10)	0.0278 (10)	0.0274 (9)	−0.0017 (7)	0.0063 (7)	−0.0026 (7)
C8	0.0273 (10)	0.0284 (10)	0.0325 (10)	0.0003 (8)	0.0038 (8)	0.0007 (8)
C9	0.0310 (11)	0.0264 (10)	0.0288 (9)	−0.0011 (8)	0.0048 (8)	0.0018 (7)

### Geometric parameters (Å, °)

**Table:**

N1—C5	1.341 (3)	C4—C5	1.387 (3)
N1—C1	1.347 (2)	C4—H4	0.9500
N2—C6	1.283 (2)	C5—H5	0.9500
N2—C7	1.429 (2)	C6—H6	0.9500
C1—C2	1.396 (3)	C7—C8	1.401 (2)
C1—H1	0.9500	C7—C9	1.408 (3)
C2—C3	1.389 (3)	C8—C9^i^	1.396 (3)
C2—C6	1.476 (3)	C8—H8	0.9500
C3—C4	1.387 (3)	C9—C8^i^	1.396 (3)
C3—H3	0.9500	C9—H9	0.9500
			
C5—N1—C1	116.01 (18)	N1—C5—H5	118.1
C6—N2—C7	118.98 (16)	C4—C5—H5	118.1
N1—C1—C2	124.86 (18)	N2—C6—C2	122.54 (17)
N1—C1—H1	117.6	N2—C6—H6	118.7
C2—C1—H1	117.6	C2—C6—H6	118.7
C3—C2—C1	117.39 (18)	C8—C7—C9	118.75 (16)
C3—C2—C6	121.57 (17)	C8—C7—N2	117.70 (16)
C1—C2—C6	121.03 (17)	C9—C7—N2	123.50 (16)
C4—C3—C2	118.94 (19)	C9^i^—C8—C7	120.79 (16)
C4—C3—H3	120.5	C9^i^—C8—H8	119.6
C2—C3—H3	120.5	C7—C8—H8	119.6
C5—C4—C3	119.1 (2)	C8^i^—C9—C7	120.45 (17)
C5—C4—H4	120.5	C8^i^—C9—H9	119.8
C3—C4—H4	120.5	C7—C9—H9	119.8
N1—C5—C4	123.7 (2)		
			
C5—N1—C1—C2	0.2 (3)	C3—C2—C6—N2	176.31 (18)
N1—C1—C2—C3	−0.5 (3)	C1—C2—C6—N2	−4.7 (3)
N1—C1—C2—C6	−179.58 (18)	C6—N2—C7—C8	−145.41 (17)
C1—C2—C3—C4	0.5 (3)	C6—N2—C7—C9	37.0 (2)
C6—C2—C3—C4	179.57 (19)	C9—C7—C8—C9^i^	−1.0 (3)
C2—C3—C4—C5	−0.2 (3)	N2—C7—C8—C9^i^	−178.72 (16)
C1—N1—C5—C4	0.2 (3)	C8—C7—C9—C8^i^	1.0 (3)
C3—C4—C5—N1	−0.2 (4)	N2—C7—C9—C8^i^	178.58 (16)
C7—N2—C6—C2	−179.26 (16)		

Symmetry codes: (i) −*x*, −*y*, −*z*+1.
